# From empirical treatment to precision intervention: a multi-database bibliometric analysis of neuropsychiatric systemic lupus erythematosus (2006–2025)

**DOI:** 10.3389/fimmu.2026.1846304

**Published:** 2026-06-03

**Authors:** Maiheliya Baihetiyaer, Xingyu Zhu, Jiang-Shan Wu, Xinyu Ju, Guo-Li Du

**Affiliations:** 1 State Key Laboratory of Pathogenesis, Prevention, and Treatment of High-Incidence Diseases in Central Asia, First Affiliated Hospital of Xinjiang Medical University, Urumqi, Xinjiang, China; 2 Department of Endocrinology, First Affiliated Hospital of Xinjiang Medical University, Urumqi, China; 3 Department of Laboratory Medicine, The First Hospital of China Medical University, Shenyang, China; 4 Department of Biochemistry and Molecular Biology, School of Laboratory Medicine, Bengbu Medical University, Bengbu, Anhui, China

**Keywords:** bibliometric analysis, biomarkers, neuropsychiatric systemic lupus erythematosus, precision medicine, targeted therapy

## Abstract

**Objective:**

Neuropsychiatric systemic lupus erythematosus (NPSLE) is a severe and complex complication of systemic lupus erythematosus that affects diagnostic decision-making and treatment strategies. However, the temporal evolution of evidence-based diagnostic and therapeutic paradigms, as well as persistent clinical bottlenecks, has not been fully characterized.

**Methods:**

We conducted a comprehensive bibliometric analysis of 1,569 NPSLE-related publications retrieved from the Web of Science Core Collection and Scopus between 2006 and 2025. In addition, relevant clinical trials from PubMed were incorporated to support an evidence-based synthesis.

**Results:**

Over the past two decades, annual publication volume in the NPSLE field increased, with an average growth rate of 6.04%. China and the United States emerged as the leading global contributors. Collaboration patterns differed across countries: the United States had a multiple-country publication (MCP) rate of 23.6%, whereas China had a lower MCP rate of 7.5%. Keyword evolution indicated a paradigm shift toward precision diagnostics and therapeutics, with neuroimaging and cognitive assessment emerging as prominent research hotspots.

**Conclusions:**

Despite substantial advances, the field remains constrained by major challenges, including clinical heterogeneity, the lack of standardized diagnostic thresholds, and the systematic exclusion of patients with active NPSLE from many SLE clinical trials. Future work should prioritize standardized diagnostic frameworks, NPSLE-specific clinical trials, and the development of effective CNS-penetrant therapies to improve long-term patient outcomes.

## 1 Introduction

Neuropsychiatric systemic lupus erythematosus (NPSLE) is among the most severe complications of systemic lupus erythematosus (SLE) (1). It presents with a highly heterogeneous spectrum of clinical manifestations, ranging from cognitive impairment and psychiatric symptoms to seizures and meningitis or encephalopathy. The clinical burden of NPSLE extends beyond the management of acute episodes, with profound and sustained long-term cognitive deterioration and substantial deterioration in patients’ everyday functioning and well-being. Despite diagnostic and therapeutic advancements over the past two decades, NPSLE management remains hindered by three core challenges: the profound heterogeneity and non-specificity of clinical symptoms; the absence of consensus diagnostic cutoffs and unified interpretive frameworks; and the limited representation of patients with active NPSLE in many pivotal SLE clinical trials. Consequently, there is a critical dearth of high-quality, evidence-based therapeutic strategies tailored specifically to this population (2–4).

To address these gaps, combining bibliometric and topic modeling methodologies can systematically characterize the evolution of NPSLE research, thereby objectively identifying the field’s foundational origins, emerging hotspots, and existing evidence gaps (5, 6). Accordingly, based on literature retrieved from the Web of Science Core Collection and Scopus databases between 2006 and 2025, this study integrates BERTopic modeling with multidimensional bibliometric tools to map the knowledge architecture and thematic trajectories of NPSLE research. By consolidating pivotal clinical evidence, this work aims to provide an objective reference framework to guide the standardization of NPSLE diagnostics, the development of mechanism-driven stratified therapies, and the rational design of future clinical trials (6).

## 2 Materials and methods

### 2.1 Data sources and data cleaning

To ensure the robustness of our findings, literature was systematically retrieved from two major academic databases: the Web of Science Core Collection (WoSCC) and Scopus. In addition, PubMed was searched for related clinical trials. For WoSCC and Scopus, the search timeframe was from January 1, 2006, to December 31, 2025, and the final literature search was performed on January 16, 2026. For PubMed, the timeframe was from January 1, 1990, to December 31, 2025, and the final search was performed on March 14, 2026. Inclusion criteria were restricted to English-language publications, and document types were limited to original research articles and reviews (7, 8). For PubMed, document types were restricted to clinical trials.

**Figure 1:**
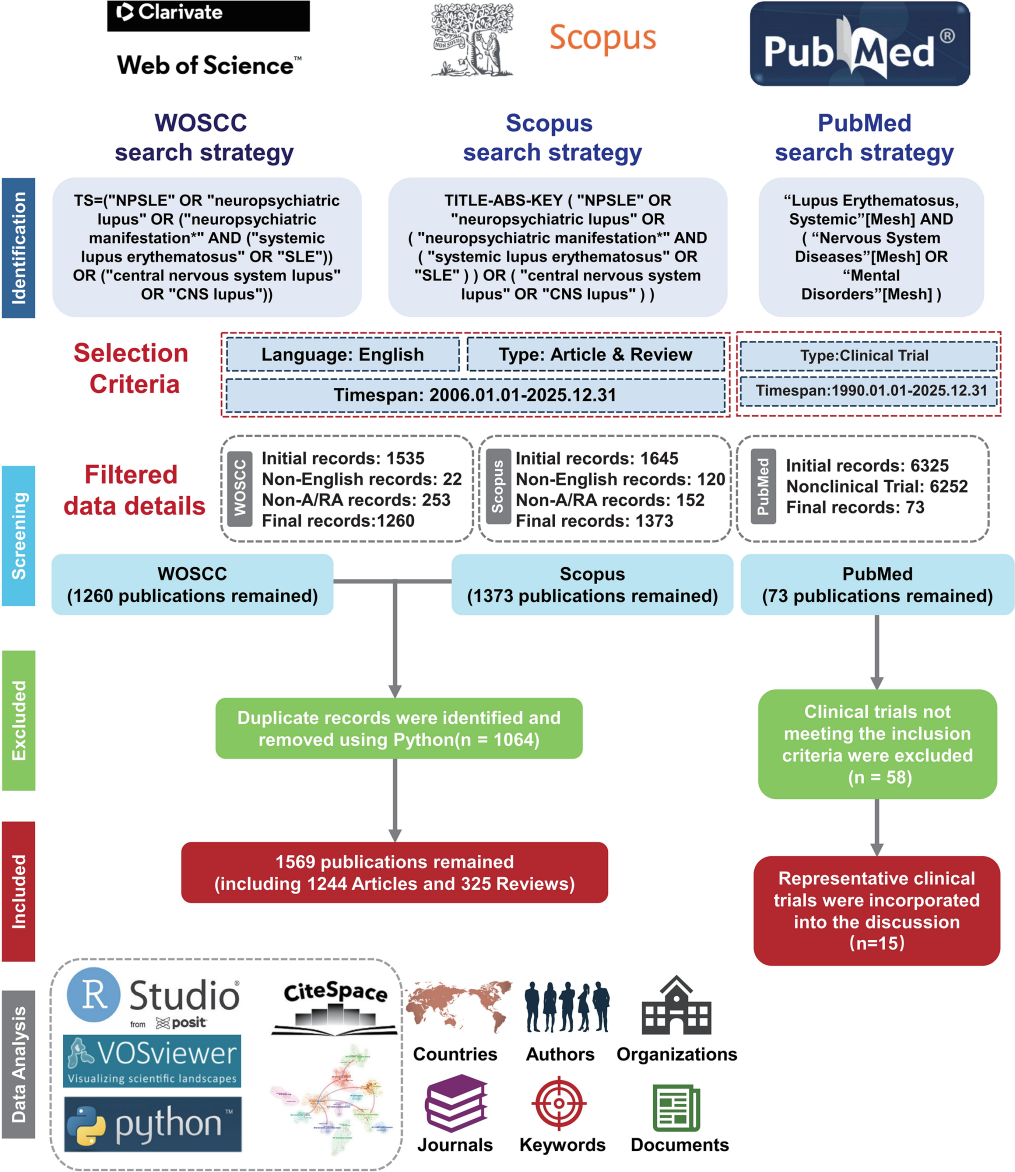
PRISMA flow diagram of literature screening and inclusion process. This workflow illustrates the systematic literature retrieval, eligibility screening, and data analysis process across three academic databases for this study. Identification: Database-specific search strategies were applied to Web of Science Core Collection (WoSCC), Scopus, and PubMed, with standardized filtering criteria for language, document type, and time period as indicated. Screening: Initial records were retrieved from WoSCC (n=1535), Scopus (n=1645), and PubMed (n=6325). After applying pre-specified exclusion criteria, 1260 records remained in WoSCC, 1373 in Scopus, and 73 in PubMed. Inclusion: Duplicate records between WoSCC and Scopus were identified and removed using custom Python scripts (n=1064), resulting in 1569 unique publications (1244 original research articles and 325 reviews) for the bibliometric analysis. For clinical evidence synthesis, 58 ineligible clinical trials were excluded from PubMed, and 15 representative high-quality studies were incorporated into the discussion. Data Analysis: Multiple validated software tools were used for bibliometric statistics, network construction, topic modeling, and visualization as listed.

To ensure methodological consistency across different database systems, we clearly distinguished the search fields and terminology standards used in each database: specifically, WoSCC was searched using the Topic Search field (TS), whereas Scopus was searched using the Title/Abstract/Keywords field (ABS), and PubMed was searched using the Medical Subject Headings (MeSH) terms. The search strategy combined Medical Subject Headings (MeSH) terms and supplementary keywords related to neuropsychiatric lupus using Boolean operators. For PubMed, the search was conducted using MeSH terms (with additional keyword terms as needed); the complete database-specific query strings are provided in **Supplementary Table 1**: WoSCC: TS = (“NPSLE” OR “neuropsychiatric lupus” OR (“neuropsychiatric manifestation” AND (“systemic lupus erythematosus” OR “SLE”)) OR (“central nervous system lupus” OR “CNS lupus”)), Scopus: TITLE-ABS-KEY (“NPSLE” OR “neuropsychiatric lupus” OR (“neuropsychiatric manifestation” AND (“systemic lupus erythematosus” OR “SLE”)) OR (“central nervous system lupus” OR “CNS lupus”)). The initial search yielded 1,260 records from WoSCC and 1,373 from Scopus. For PubMed, an initial 6,325 records were retrieved, and 73 records remained after restricting to clinical trials (for further screening). Ultimately, 1,569 unique publications were included in the final analysis, consisting of 1,244 research articles and 325 reviews (**Figure 1**). Clinical trials were screened independently by two investigators, and discrepancies were resolved by a third reviewer. In addition, we included a consistency check, yielding an inter-reviewer agreement of κ = 0.89 (95% CI: 0.86–0.92), which strengthens the reliability and methodological rigor of the screening process. The pre-specified inclusion and exclusion criteria are provided in **Supplementary Table 7**. Among the PubMed-retrieved clinical trials, 58 were excluded after full-text screening, and 15 representative trials were incorporated into the discussion (**Supplementary Table 1**).

### 2.2 Bibliometric analysis

We used bibliometrix (v5.0) in R (v4.4.5) (9) to compute basic bibliometric statistics, including annual publication volume, core authors, journal distribution, and citation-related indicators. During the import into the bibliometrix R package, we applied the package’s disambiguation/standardization functions to normalize bibliographic records by removing non-alphabetic characters, punctuation marks, and redundant spaces, thereby reducing noise introduced by cross-database formatting differences. Prior to import, duplicate records across WoSCC and Scopus were identified and removed using custom Python scripts during data integration, ensuring that subsequent disambiguation/standardization and bibliometric analyses were conducted on harmonized unique records. Thematic evolution was explored using bibliometrix’s built-in thematic evolution function. For collaboration network construction (authors, countries, and institutions), we used VOSviewer (v1.6.20). Communities were identified using the VOS clustering approach together with modularity optimization. Network thresholds were set as follows: the minimum number of publications was 5 and the minimum number of citations was 0. CiteSpace (v6.2.R1) was used for keyword clustering, keyword burst detection, and citation burst analysis. Time slicing was set to 1-year intervals. Node types included authors, institutions, and keywords. Network pruning was performed using Pathfinder and pruning sliced networks. Key parameters were set as: g-index (k = 25), LRF = 2.5, L/N = 10, LBY = 5, and e = 1.0. Finally, outputs were visualized in Python (v3.14) using pandas, matplotlib, and seaborn (10–12).

## 3 Results

### 3.1 Publication trends and geographical distribution

**Figure 2:**
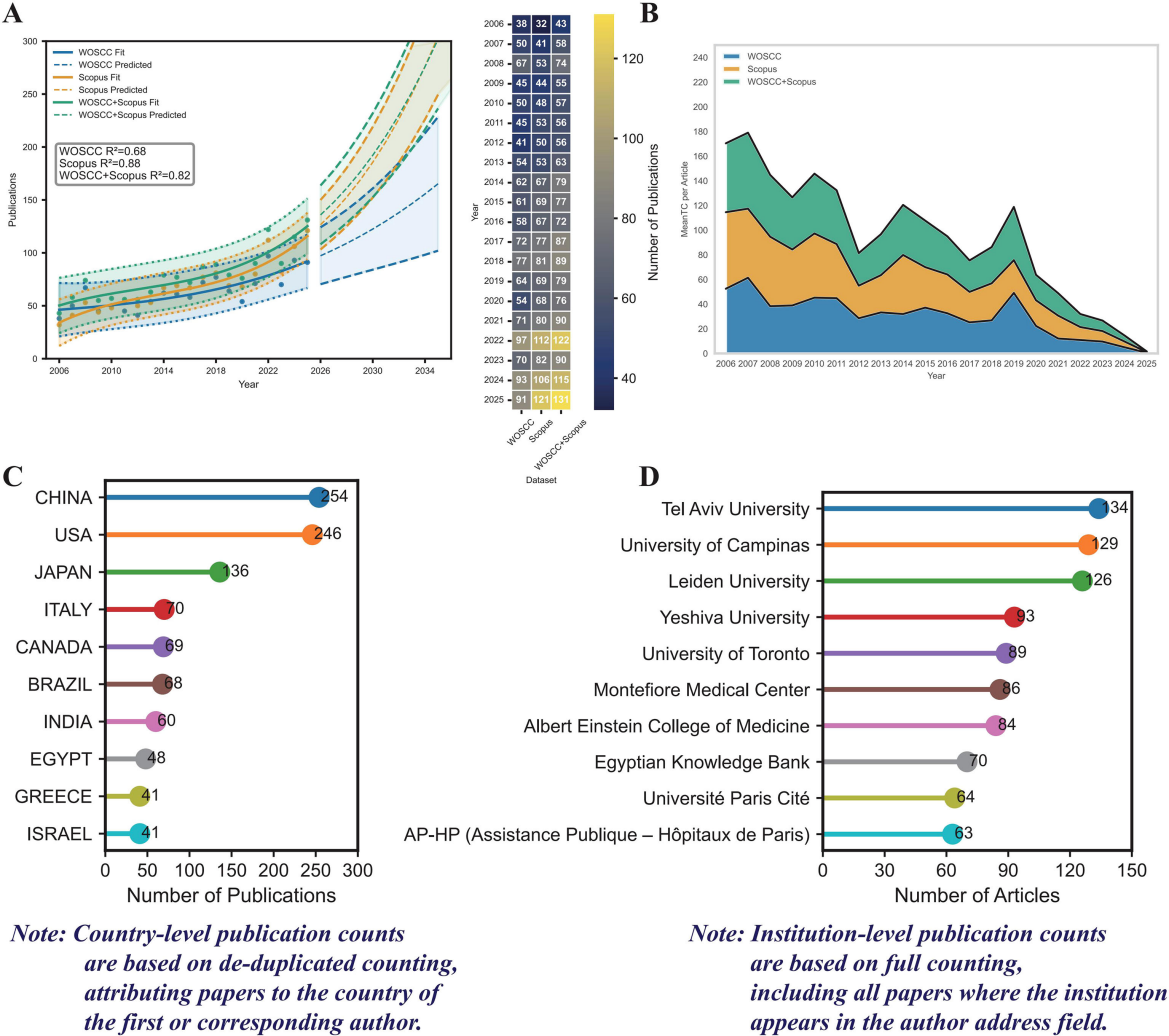
Publication trends and geographical distribution of NPSLE-related publications (2006–2025). **(A)** Annual publication volume and growth fitting curves of NPSLE-related literature in WoSCC, Scopus, and the combined database, with R2 values indicating the goodness of fit for each trend line; **(B)** Annual citation volume analysis across WoSCC, Scopus, and the combined database; **(C)** Lollipop chart of the top 10 countries by total publication output. Country-level publication counts use de-duplicated counting, attributing each paper only to the country of the first or corresponding author to avoid overcounting international collaborative studies; **(D)** Lollipop chart of the top 10 institutions by total publication output. Institution-level publication counts use full counting, including all papers where the institution appears in any author address field; Citation volume patterns were broadly comparable across databases, indicating consistent scholarly recognition of NPSLE research regardless of indexing source.

A total of 1,569 publications met the inclusion criteria and were included in the final analysis (**Table 1**). Analysis of the combined dataset showed an average annual growth rate of 6.04% in publication volume from 2006 to 2025. This growth was more pronounced in the Scopus database (7.25%) compared to the WoSCC database (4.7%), a difference likely attributable to the broader indexing scope of Scopus. Furthermore, curve fitting indicated an accelerated growth trend in overall literature output across the combined databases (R² = 0.82) (**Figure 2**, **Table 1**). Citation volume patterns were broadly comparable across databases, indicating consistent scholarly recognition of NPSLE research regardless of indexing source.

**Table 1:** Overview of publication metrics across databases (WoSCC, Scopus, and combined dataset).

Description	WOSCC	Scopus	WOSCC+Scopus
Timespan	2006:2025	2006:2025	2006:2025
Sources (journals, books, etc)	344	444	494
Documents	1260	1373	1569
Annual growth rate %	4.7	7.25	6.04
Document average age	9.28	8.71	9.03
Average citations per doc	27.06	26.3	27.55
References	30914	6632	32941
Keywords plus (ID)	2238	8145	5145
Author’s keywords (DE)	2076	2252	2488
Authors	6614	6449	7961
Authors of single-authored docs	25	36	44
Single-authored docs	27	44	50
Co-authors per Doc	7.25	6.94	6.87
International co-authorships %	19.13	17.33	17.14
article	998	1116	1244
review	262	257	325

WoSCC, Web of Science Core Collection; TC, total citations; ACR, average citations per article; AGR, average annual growth rate. Data for 2025 represent partial-year records as of the search date. This table presents a comparative overview of publication metrics for neuropsychiatric systemic lupus erythematosus (NPSLE) research (2006–2025) retrieved from the Web of Science Core Collection (WoSCC) and Scopus databases. Data for 2025 represent partial-year records as of the search date (May 1, 2025).

Geographically, publication output was highly concentrated (**Figure 2C**). China (n = 254, 16.2%), the United States (n = 246, 15.7%), and Japan (n = 136, 8.7%) were the top three contributing nations (**Figure 2D**). Collectively, the top ten countries accounted for 65.9% of the total global output (**Figure 2E**). Distinct patterns of international collaboration were observed among these leading nations. For instance, the multiple-country publication (MCP) rate for the United States was 23.6%, whereas Israel exhibited a markedly higher MCP rate of 46.3% (**Table 2**), highlighting divergent national strategies for international research cooperation. At the institutional level, Tel Aviv University (n = 134) and the University of Campinas (n = 129) showed relatively high publication volumes and citation frequencies (**Figure 2D**). Note that institutional counts use full counting, whereas national counts use de-duplicated counting; therefore, these values are not directly comparable.

**Table 2:** Top 10 most productive countries and their multiple-country publication rates.

Country	Articles	Articles %	SCP	MCP	MCP %
CHINA	254	16.2	235	19	7.5
USA	246	15.7	188	58	23.6
JAPAN	136	8.7	132	4	2.9
ITALY	70	4.5	60	10	14.3
CANADA	69	4.4	38	31	44.9
BRAZIL	68	4.3	61	7	10.3
INDIA	60	3.8	57	3	5
EGYPT	48	3.1	46	2	4.2
GREECE	41	2.6	24	17	41.5
ISRAEL	41	2.6	22	19	46.3

MCP, multiple-country publication; SCP, single-country publication; MCP ratio, proportion of publications involving international collaboration (calculated as MCP/total publications). Countries are ranked by total publication count. This table lists the top 10 countries by total publication output in NPSLE research (2006–2025). Countries are ranked in descending order of total number of articles.

### 3.2 Analysis of author, country, and institutional output

**Figure 3:**
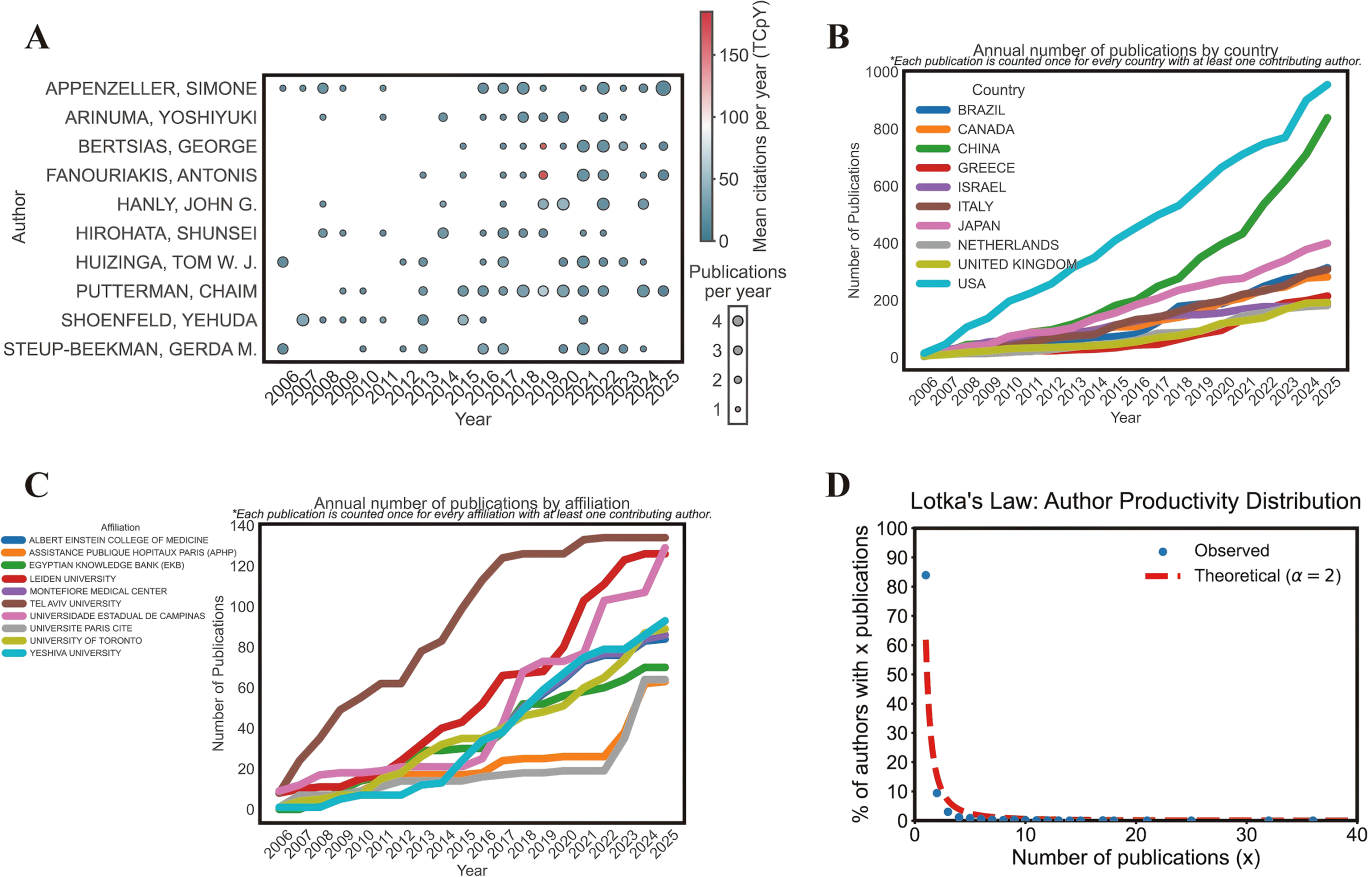
Productivity analysis of authors, institutions, and countries in the NPSLE field (2006–2025). **(A)** Annual publication output of the top 10 core authors in the field; **(B)** Annual publication output of the top 3 leading countries (China, the United States, Japan); **(C)** Annual publication output of the top 5 leading institutions; **(D)** Fitting curve of author productivity distribution based on Lotka’s Law, revealing the skewed productivity pattern of authors in the NPSLE field.

To further characterize the research landscape of NPSLE from 2006 to 2025, we analyzed the productivity and output patterns of key authors, countries, and institutions (**Figure 3**, **Table 3**). At the individual author level, Chaim Putterman (n = 36), Simone Appenzeller (n = 32), and Gerda M. Steup-Beekman (n = 25) were the top contributors by publication volume (**Figure 3A**, **Table 3**). These authors also demonstrated substantial academic impact, with h-indices of 22, 18, and 16, and total citation counts of 1,666, 907, and 904, respectively (**Table 3**).

**Table 3:** Top 10 authors by academic impact metrics (publications, citations, h-index).

Author	h_index	g_index	m_index	TC	NP	PY_start
PUTTERMAN CHAIM	22	36	1.222	1666	36	2009
APPENZELLER SIMONE	18	30	0.857	907	32	2006
STEUP-BEEKMAN GERDA M.	16	25	0.762	904	25	2006
HIROHATA SHUNSEI	14	17	0.737	788	17	2008
HUIZINGA TOM W. J.	14	21	0.667	684	21	2006
SHOENFELD YEHUDA	13	17	0.65	1513	17	2007
VAN BUCHEM MARK A.	13	15	0.619	666	15	2006
DIAMOND BETTY	12	12	0.571	1123	12	2006
HANLY JOHN G.	12	17	0.632	920	17	2008
BERTSIAS GEORGE	11	18	0.917	1831	18	2015

TC, total citations; ACR, average citations per article; h-index, Hirsch index. Authors are ranked by total publication count. This table presents the top 10 authors in NPSLE research (2006–2025) ranked by total number of publications. All metrics were retrieved from the Web of Science Core Collection.

At the national level, China and the United States exhibited the most rapid growth in annual publication output, consistently leading global contributions (**Figure 3B**). As previously noted, China (n = 254), the United States (n = 246), and Japan (n = 136) were the top three countries by cumulative publication volume. Institutionally, Tel Aviv University, Leiden University, and Albert Einstein College of Medicine demonstrated a steady increase in annual publication volume, serving as the primary affiliations for the field’s leading scholars (**Figure 3C**).

Finally, an evaluation of overall author productivity using Lotka’s Law (**Figure 3D**) revealed a highly skewed distribution typical of bibliometric data: a small fraction of core authors contributed the majority of the literature, while the vast majority of authors produced only one or two publications.

### 3.3 Keyword co-occurrence and hotspot evolution

**Figure 4:**
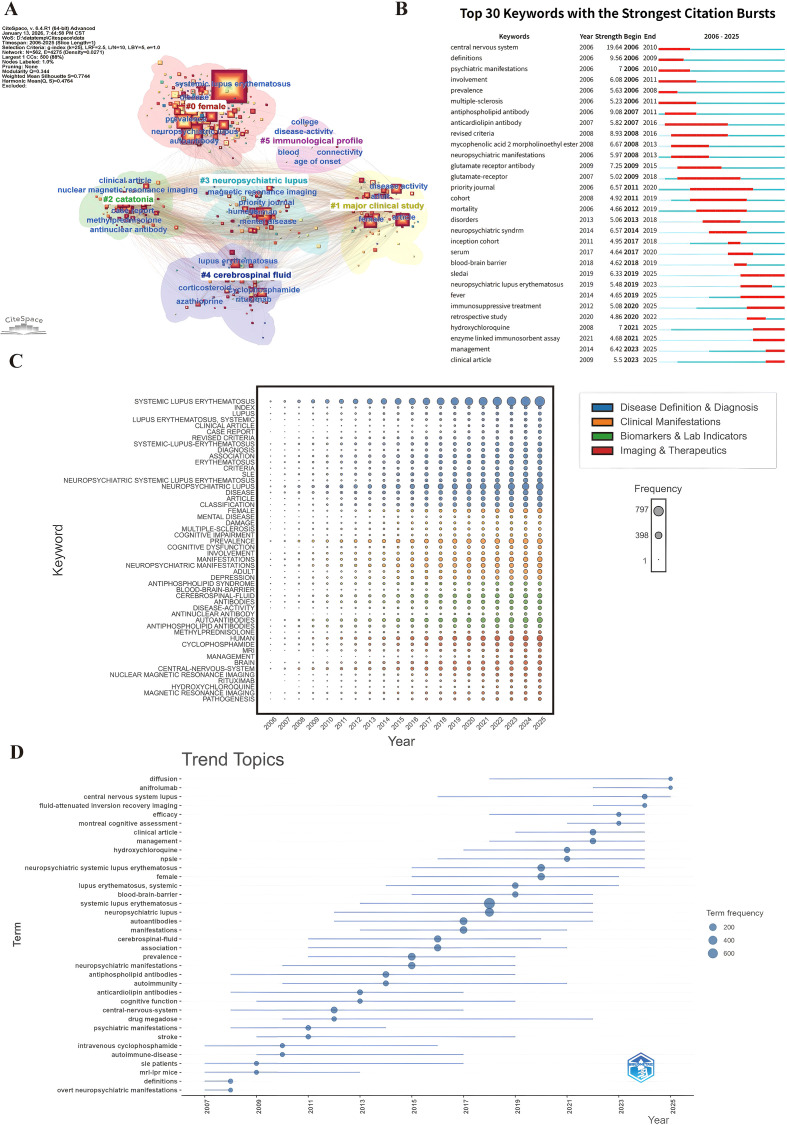
Keyword co-occurrence and hotspot evolution analysis of NPSLE research. **(A)** Clustering network map of high-frequency keywords, with 5 primary thematic clusters identified via co-occurrence association; **(B)** Top 30 keywords with the strongest citation bursts over the 20-year study period, with the red bar indicating the duration of keyword burst, “burst strength” reflects the magnitude of the sudden increase in keyword usage over time, and a longer red bar indicates a longer burst duration; **(C)** Cluster heatmap of annual frequency changes for high-frequency keywords, categorized into four core thematic domains, color intensity represents the annual frequency of keywords within each thematic domain (darker indicates higher frequency); **(D)** Trend graph of representative keywords with significant temporal frequency changes from 2006 to 2025, steeper rises indicate accelerating research attention for the corresponding keywords over time. The co-occurrence network partitions NPSLE research into five thematic clusters. Citation-burst analysis further indicates a temporal shift: early bursts (2006–2012) emphasize disease definition and epidemiology, whereas sustained bursts from 2019 to 2025 concentrate on precision diagnostics and therapeutic strategies.

To map the thematic architecture and evolving priorities within the NPSLE domain from 2006 to 2025, we conducted a keyword co-occurrence analysis (**Figure 4**; **Supplementary Table 2**). The most frequent keywords were “systemic lupus erythematosus” (n = 886), “neuropsychiatric lupus” (n = 396), and “human” (n = 258), forming the foundational lexicon of the field. Based on co-occurrence frequencies, the keyword network was partitioned into five primary thematic clusters: major clinical studies, biomarkers, neuropsychiatric lupus, cerebrospinal fluid analysis, and immunological characteristics. This clustering captures the overarching thematic framework and intrinsic structural linkages of NPSLE research (**Figure 4A**).

Keyword prominence evolved significantly over the study period. Burst analysis of the top 30 keywords (**Figure 4B**) revealed a clear temporal shift in research focus. Early-burst keywords were predominantly associated with fundamental disease characteristics and epidemiology, such as “central nervous system,” “psychiatric manifestations,” and “prevalence.” In contrast, keywords with sustained bursts from 2019 to 2025 shifted toward precision diagnostics and therapeutics, including “neuropsychiatric lupus erythematosus,” “immunosuppressive treatment,” and “hydroxychloroquine.” This transition reflects the field’s progression from foundational disease characterization toward targeted therapeutic intervention.

Annual fluctuations in keyword popularity further illustrate this evolutionary trajectory (**Figure 4C**). Core keywords were categorized into four primary domains: disease definition and diagnosis, clinical manifestations, biomarkers and laboratory indices, and imaging and therapeutics. A detailed temporal analysis of trending terms (**Figure 4D**) confirmed that the 2006–2012 period was dominated by definitional terms, such as “overt neuropsychiatric manifestations,” “definitions,” and “autoimmune disease.” Conversely, from 2013 to 2025, terms related to diagnostic evaluation and pharmacotherapy—such as “diffusion,” “infliximab,” “Montreal Cognitive Assessment,” and “efficacy”—exhibited marked increases in frequency, highlighting the emergence of novel clinical and investigative priorities.

### 3.4 Author, country, and institutional collaboration networks

**Figure 5:**
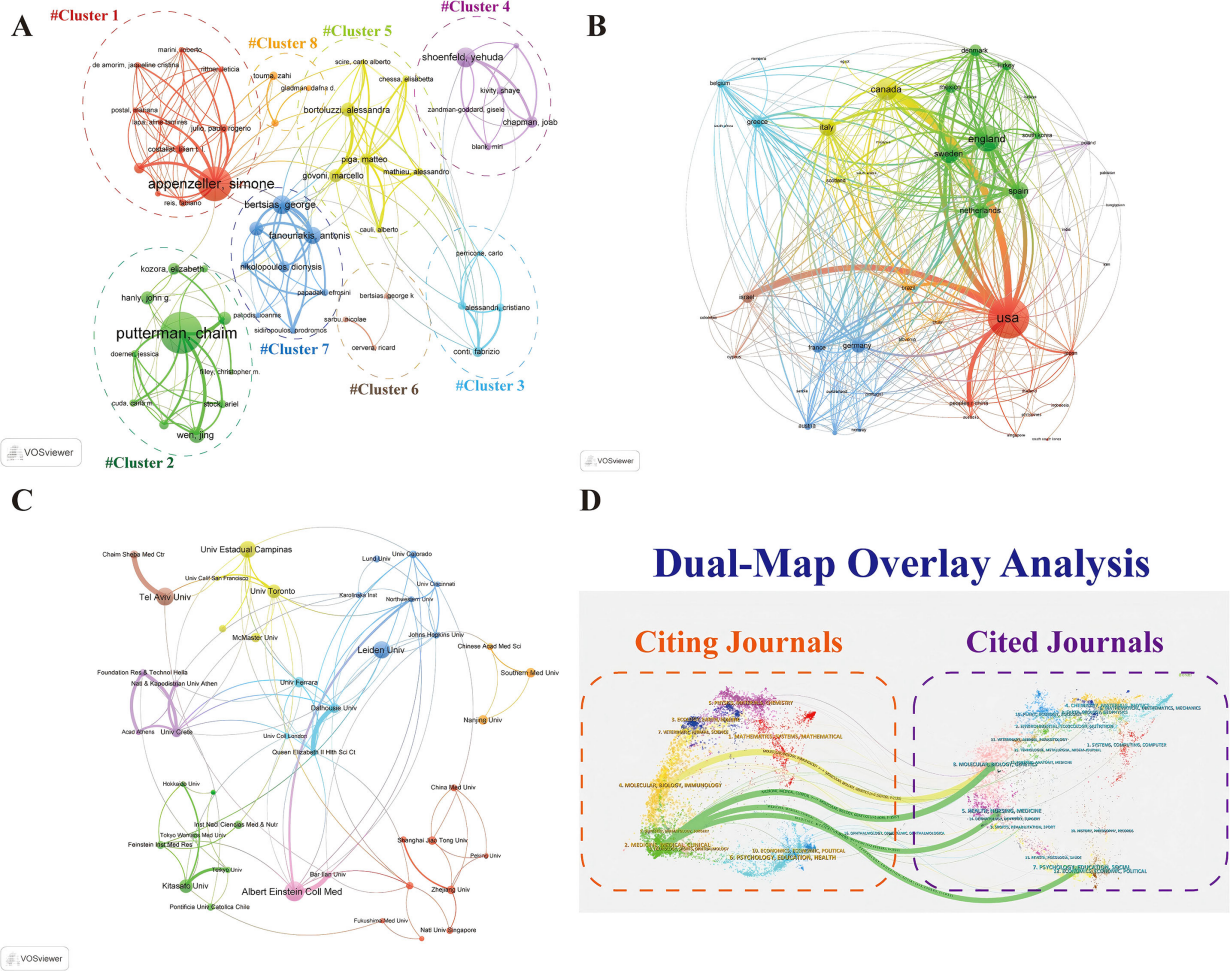
Collaboration network analysis among authors, countries, and institutions. **(A)** Collaboration network of core authors in the NPSLE field, with 8 distinct collaborative clusters identified, nodes represent authors; node size reflects citation impact, and edge thickness represents co-authorship strength/association between authors. Node colors denote detected collaboration communities. **(B)** International collaboration network between countries, with node size corresponding to total publication volume and line thickness corresponding to collaboration strength, Nodes represent countries; larger nodes indicate higher publication volume, and thicker links reflect stronger co-authorship collaboration strength; **(C)** Collaboration network of global core research institutions, nodes represent institutions; node size reflects publication/citation volume, and edge thickness indicates collaboration strength; **(D)** Dual-map overlay analysis of journals, characterizing the interdisciplinary knowledge flow of NPSLE research (left: citing journals; right: cited journals).

Analysis of author collaboration networks partitioned the core authors into eight distinct clusters based on co-occurrence linkages (**Figure 5A**). As detailed in **Supplementary Table 3**, George K. Bertsias (1,831 citations), Chaim Putterman (1,666 citations), and Yehuda Shoenfeld (1,513 citations) received the highest total citations. In contrast, Gerda M. Steup-Beekman (total link strength [TLS] = 96), Tom W. J. Huizinga (TLS = 86), and Simone Appenzeller (TLS = 76) led in collaborative network breadth. This distinction highlights variations among core authors regarding overall citation impact versus collaborative engagement.

At the national level, the international collaboration network (**Figure 5B**) identified the United States as a primary hub, maintaining extensive transnational collaborations with Italy, the United Kingdom, and Canada. The United States (12,662 citations), Italy (6,548 citations), and the United Kingdom (6,115 citations) ranked highest in total citations (**Supplementary Table 4**). Furthermore, the United States (TLS = 258), the United Kingdom (TLS = 161), and Canada (TLS = 140) exhibited the highest total link strengths, underscoring their central roles in global research networks.

Institutional collaboration analysis (**Figure 5C**) demonstrated that Tel Aviv University (2,184 citations), Leiden University (1,892 citations), and Albert Einstein College of Medicine (1,657 citations) accrued the most citations (**Supplementary Table 5**). Conversely, the University of Crete (TLS = 33), Albert Einstein College of Medicine (TLS = 31), and Tel Aviv University (TLS = 18) displayed the strongest collaborative linkages.

Finally, a dual-map overlay of journals (**Figure 5D**) characterized the interdisciplinary knowledge flow in NPSLE research. Citing articles were primarily published in molecular biology, immunology, and rheumatology journals (e.g., Lupus, Arthritis and Rheumatology), whereas the cited references were broadly distributed across general medicine, pharmacology, and neuroscience journals (e.g., Neurology, Brain).

### 3.5 Journal distribution and high-impact literature

The dissemination of research findings in this field demonstrates pronounced journal concentration (**Table 4**). Lupus ranked first with 218 articles, 5,523 total citations, and an h-index of 41, establishing it as the primary publication venue and the most influential specialized journal in the NPSLE domain. Annals of the Rheumatic Diseases followed closely, garnering 4,470 total citations and an h-index of 18. Furthermore, leading rheumatology and immunology journals, including Autoimmunity Reviews, Rheumatology, and The Journal of Rheumatology, served as core publication platforms, with all top ten journals maintaining an h-index above 14.

**Table 4:** Top 10 journals by publication volume, citations, and h-index.

Source	h_index	g_index	m_index	TC	NP	PY_start
LUPUS	41	59	1.952	5523	218	2006
AUTOIMMUNITY REVIEWS	25	33	1.25	2611	33	2007
RHEUMATOLOGY	20	38	1	1480	41	2007
JOURNAL OF RHEUMATOLOGY	19	25	0.905	881	25	2006
ANNALS OF THE RHEUMATIC DISEASES	18	22	0.857	4470	22	2006
ARTHRITIS AND RHEUMATISM	17	17	0.81	1619	17	2006
RHEUMATOLOGY INTERNATIONAL	17	27	0.85	748	30	2007
CLINICAL RHEUMATOLOGY	15	25	0.714	665	34	2006
ARTHRITIS RESEARCH and THERAPY	14	25	0.667	713	25	2006
FRONTIERS IN IMMUNOLOGY	14	27	1	743	34	2013

TC, total citations; ACR, average citations per article; h-index, Hirsch index; IF, impact factor (2025). Journals are ranked by publication count. This table lists the top 10 journals publishing NPSLE research (2006–2025) ranked by total number of publications. All metrics were retrieved from the Web of Science Core Collection.

**Figure 6:**
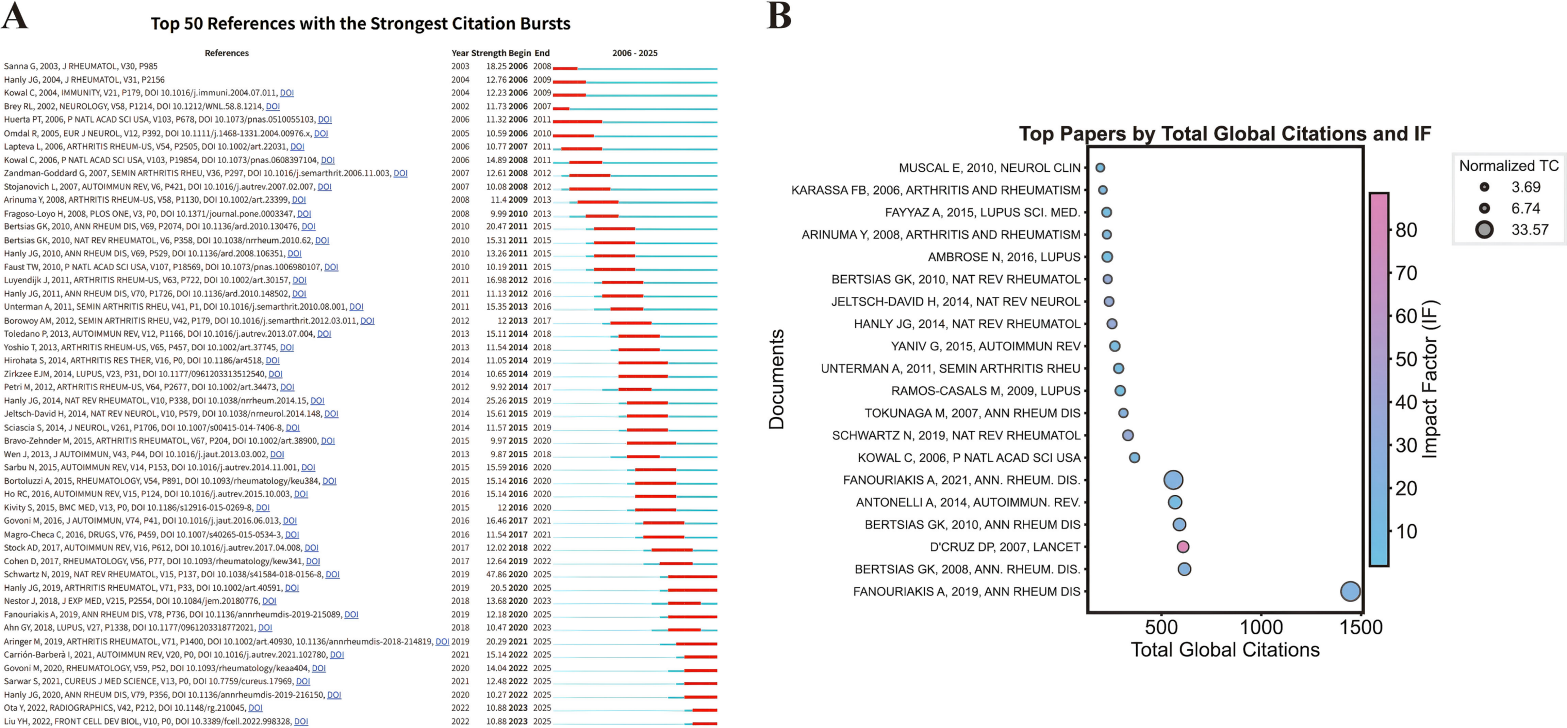
Citation analysis of NPSLE-related literature. **(A)** Top 50 references with the strongest citation bursts from 2006 to 2025, with the red bar indicating the duration of citation burst; **(B)** Bubble chart of the top 20 most-cited publications worldwide, with bubble size corresponding to total citation counts and color corresponding to the 2025 impact factor (IF) of the publishing journal. Citation-burst stratification reveals that foundational diagnostic/definition works dominated earlier years, whereas more recent bursts (around 2019–2025) increasingly reflect mechanistic studies, biomarkers, and treatment regimens.

High-impact literature was similarly concentrated within these leading journals (**Figure 6B**, **Table 5**). The 2019 article by Fanouriakis et al., published in Annals of the Rheumatic Diseases, received the most global citations (n = 1,448) and the highest normalized total citation score (33.57), indicating an academic impact substantially above the field average. The research group led by G. K. Bertsias contributed four articles to the top 20, representing the highest concentration of high-impact research output by a single team. Additionally, highly cited literature frequently appeared in high-impact general medical and review journals. For instance, the 2007 article by D’Cruz et al. was published in The Lancet, which possessed a 2025 impact factor of 88.5.

**Table 5:** Top 20 most cited articles in NPSLE research.

Paper	Global total citations	Normalized TC	IF(2025)
FANOURIAKIS A, 2019, ANN RHEUM DIS	1448	33.57	20.6
BERTSIAS GK, 2008, ANN. RHEUM. DIS.	615	12.27	20.6
D’CRUZ DP, 2007, LANCET	608	9.87	88.5
BERTSIAS GK, 2010, ANN RHEUM DIS	590	12.17	20.6
ANTONELLI A, 2014, AUTOIMMUN. REV.	568	14.00	8.3
FANOURIAKIS A, 2021, ANN. RHEUM. DIS.	560	30.81	20.6
KOWAL C, 2006, P NATL ACAD SCI USA	365	6.54	9.1
SCHWARTZ N, 2019, NAT REV RHEUMATOL	332	7.70	32.7
TOKUNAGA M, 2007, ANN RHEUM DIS	309	5.02	20.6
RAMOS-CASALS M, 2009, LUPUS	293	6.94	1.9
UNTERMAN A, 2011, SEMIN ARTHRITIS RHEU	285	6.54	4.4
YANIV G, 2015, AUTOIMMUN REV	266	7.06	8.3
HANLY JG, 2014, NAT REV RHEUMATOL	252	6.21	32.7
JELTSCH-DAVID H, 2014, NAT REV NEUROL	237	5.84	33.1
BERTSIAS GK, 2010, NAT REV RHEUMATOL	230	4.74	32.7
AMBROSE N, 2016, LUPUS	228	7.38	1.9
ARINUMA Y, 2008, ARTHRITIS AND RHEUMATISM	226	4.51	10.9
FAYYAZ A, 2015, LUPUS SCI. MED.	225	5.97	3.5
KARASSA FB, 2006, ARTHRITIS AND RHEUMATISM	206	3.69	10.9
MUSCAL E, 2010, NEUROL CLIN	193	3.98	2.9

TC, total citations (global); NCTC, normalized total citation score; IF, impact factor (2025). Articles are ranked by global citation count. This table presents the top 20 most-cited articles in the field of neuropsychiatric systemic lupus erythematosus (NPSLE) research published between 2006 and 2025, ranked in descending order of global total citations. All citation data were retrieved from the Web of Science Core Collection.

Citation burst analysis of the referenced literature revealed a distinct temporal stratification (**Figure 6A**). Among the top 50 articles with the highest citation burst strengths, the early phase (2006–2012) was dominated by foundational literature establishing disease definitions and diagnostic criteria, which exhibited prolonged burst durations. In contrast, the recent phase (2019–2025) was characterized by studies detailing immune mechanisms, biomarkers, and therapeutic regimens. Specifically, the 2019 publication by Hanly et al. achieved the highest burst strength (47.88) across the evaluated timeframe. Furthermore, a relative hiatus in strong citation bursts was observed between 2013 and 2018. This period coincides with the transitional phase previously identified, during which the field’s research focus shifted from fundamental disease characterization to targeted therapeutic interventions.

### 3.6 Thematic clustering, attention dynamics, and evolutionary trajectories

**Figure 7:**
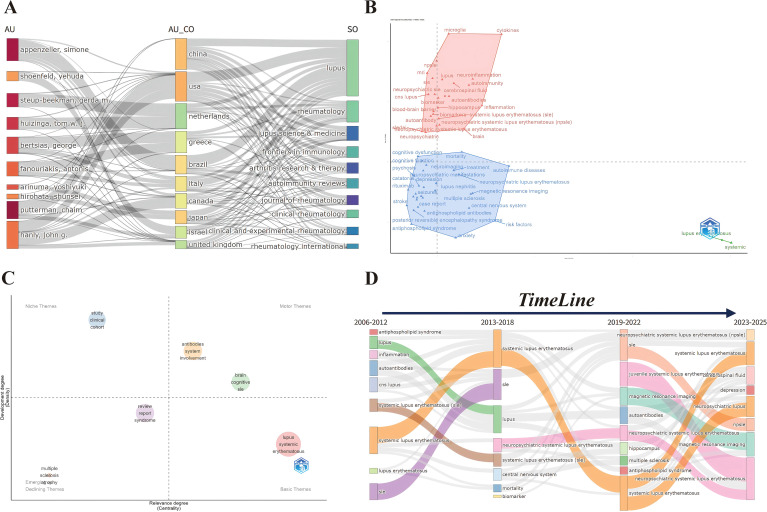
Thematic clustering and evolutionary trajectory analysis of NPSLE research. **(A)** Three-way Sankey diagram showing the association network among core authors, affiliated countries, and publishing journals; **(B)** Multiple Correspondence Analysis (MCA) of keywords, identifying three core thematic clusters of NPSLE research; **(C)** Thematic quadrant map based on centrality and novelty of core research themes, Themes in the high-centrality/low-novelty quadrant indicate well-established but mature research areas; high-centrality/high-novelty indicates active emerging directions with strong relevance to the field; low-centrality/high-novelty suggests novel but relatively less connected themes; and low-centrality/low-novelty reflects niche topics with limited recent activity; **(D)** Thematic evolution timeline delineating chronological shifts in core research themes over the 20-year study period. MCA identifies three major thematic clusters corresponding to (i) neuroinflammation/autoimmune mechanisms, (ii) cognitive dysfunction/neuroimaging, and (iii) antiphospholipid syndrome/biomarkers. The quadrant plot differentiates themes by centrality (maturity) and novelty (emerging activity), while the timeline depicts a progression from foundational characterization toward precision phenotyping and advanced diagnostic modalities.

To systematically map the thematic architecture and its evolution from 2006 to 2025, multidimensional topic modeling was employed (**Figure 7**). First, a three-way Sankey diagram illustrated the associative network among core authors, their affiliated countries, and publishing journals (**Figure 7A**). In the Sankey diagram (**Figure 7A**), the thickest flows indicate the predominant linkages: Appenzeller and Putterman are mainly connected to major hubs such as the United States and China, whereas Shoenfeld is predominantly linked to institutions in Israel. These predominant country-to-journal pathways show that the associated publications are largely disseminated through specialized lupus and rheumatology-related journals such as Lupus and Rheumatology. This highlights the established pathways between knowledge generation and dissemination among the field’s core entities.

Regarding thematic structure, Multiple Correspondence Analysis (MCA) of keywords categorized the research into three principal clusters: (1) neuroinflammation and autoimmune mechanisms, (2) cognitive dysfunction and neuroimaging, and (3) antiphospholipid syndrome and biomarkers. These clusters directly correspond to three core investigative trajectories focusing on pathogenesis, clinical evaluation, and disease phenotyping, respectively (**Figure 7B**).

A thematic quadrant map further evaluated the centrality and novelty of these research themes (**Figure 7C**). Foundational terms (e.g., “systemic lupus erythematosus”) were positioned in the high-centrality, low-novelty quadrant. Themes such as “brain” and “autoantibodies” occupied the high-centrality, high-novelty quadrant, indicating well-developed and active research areas. Conversely, terms like “clinical cohort” appeared in the low-centrality, high-novelty quadrant, while “multiple sclerosis” was located in the low-centrality, low-novelty quadrant.

Finally, a thematic evolution timeline delineated the chronological shifts in research focus over the two-decade study period (**Figure 7D**). From 2006 to 2012, core themes focused on foundational disease characteristics and pathogenic mechanisms, such as antiphospholipid syndrome and inflammation. Between 2013 and 2018, the focus transitioned toward target organ and systemic involvement, encompassing the central nervous system. From 2019 to 2022, precision phenotyping of NPSLE emerged as the central theme. Most recently (2023–2025), advanced diagnostic and evaluative modalities—specifically neuroimaging (MRI), fluid biomarkers (cerebrospinal fluid), and psychiatric assessment (depression)—have become the primary research frontiers.

### 3.7 Fine-grained topic identification and temporal trajectories via BERTopic modeling

**Figure 8:**
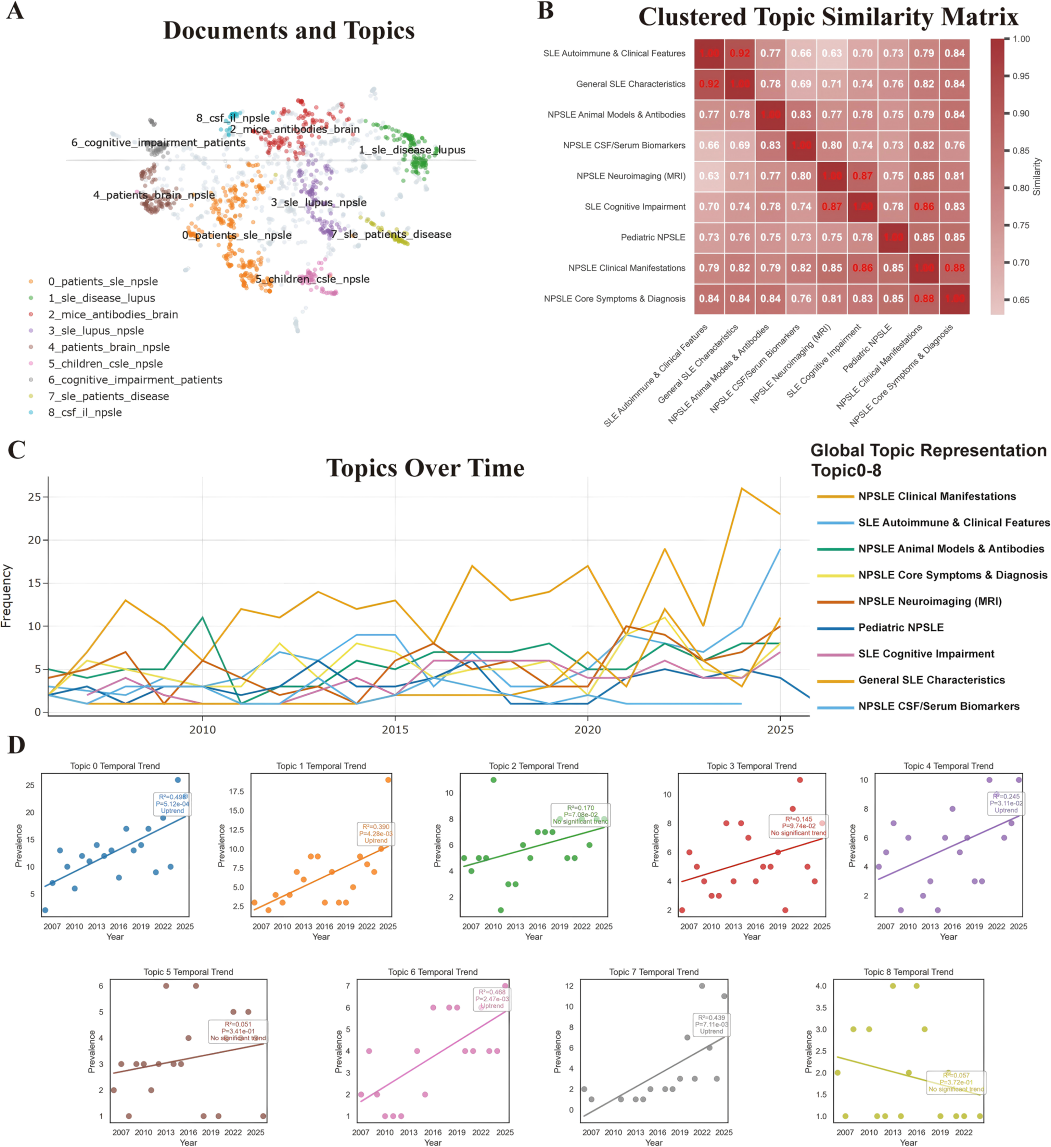
Fine-grained topic identification and temporal trend analysis via BERTopic modeling. **(A)** Document-topic projection map and intertopic distance map of the 9 core themes identified by the BERTopic model; **(B)** Inter-topic similarity matrix quantifying the correlation strength between the 9 core themes, smaller inter-topic distances/higher similarity values indicate that themes share more overlapping vocabulary and concepts; **(C)** Temporal evolution graph of the 9 core themes from 2006 to 2025; **(D)** Temporal trend regression plots for individual core themes, with linear regression results for statistical significance, a positive regression trend with statistical significance (reported R² and P values) indicates an increasing prevalence of the corresponding topic over time. Topic projections and intertopic distance/similarity matrices quantify how closely related the nine BERTopic-derived themes are, supporting the presence of coherent clinical–mechanistic research pathways.

To complement the macroscopic thematic analysis, we applied the BERTopic framework to conduct fine-grained topic modeling on the included literature. This approach delineated specific thematic classifications, inter-topic associations, and chronological trajectories from 2006 to 2025 (**Figure 8**, **Table 6**; **Supplementary Table 6**). The model successfully identified nine core themes, providing comprehensive coverage of the NPSLE research landscape (**Table 6**).

**Table:** Table 6 Summary of nine core topics identified by BERTopic modeling.

Topic ID	Topic label	Document count	Top 10 keywords (ranked by weight)
0	NPSLE Clinical Manifestations	256	patients, sle, npsle, disease, np, manifestations, lupus, neuropsychiatric, clinical, systemic
1	SLE Autoimmune and Clinical Features	121	sle, disease, lupus, autoimmune, clinical, systemic, erythematosus, manifestations, treatment, patients
2	NPSLE Animal Models and Autoantibodies	117	mice, antibodies, brain, npsle, autoantibodies, lupus, patients, bbb, neuropsychiatric, mrl/lpr
3	NPSLE Core Symptoms and Diagnosis	109	sle, lupus, npsle, neuropsychiatric, systemic, erythematosus, symptoms, patient, case, brain
4	NPSLE Neuroimaging (MRI)	102	patients, brain, npsle, mri, non-npsle, matter, sle, gyrus, wm, white
5	Pediatric NPSLE	64	children, csle, npsle, patients, pediatric, lupus, disease, jsle, diagnosis, neuropsychiatric
6	SLE Cognitive Impairment	64	cognitive, impairment, patients, sle, cd, ci, memory, moca, dysfunction, function
7	General SLE Characteristics	57	sle, patients, disease, lupus, background, npsle, systemic, clinical, autoimmune, study
8	NPSLE CSF and Serum Biomarkers	31	csf, il, npsle, serum, levels, patients, cerebrospinal, higher, fluid, controls

n, number of documents assigned to the topic. Topics are ranked by document volume. Topic labels were generated based on representative keywords and manual curation. This table presents the 9 core topics identified by the BERTopic topic modeling algorithm.

The three largest topics by document volume were “clinical manifestations of NPSLE” (n = 256), “autoimmune and clinical characteristics of SLE” (n = 121), and “NPSLE animal models and autoantibodies” (n = 117). The remaining themes encompassed core symptoms and diagnosis, neuroimaging (MRI), pediatric NPSLE, cognitive dysfunction, general SLE characteristics, and cerebrospinal fluid/serum biomarkers. Collectively, these themes represent the primary research domains of the field, spanning clinical phenotypes, fundamental mechanisms, diagnostic evaluations, and biomarker discovery.

The spatial distribution, inter-topic distances, and document affiliations of these nine themes are mapped in **Figure 8A**, illustrating the clustering patterns and boundaries of each topic. An inter-topic similarity matrix (**Figure 8B**) further quantified the associations among themes. “Autoimmune and clinical characteristics of SLE” and “general characteristics of SLE” exhibited the highest similarity (0.95). The similarity scores between “clinical manifestations of NPSLE” and both “core symptoms and diagnosis of NPSLE” and “cognitive dysfunction in SLE” were 0.85. Additionally, the similarity between “NPSLE neuroimaging (MRI)” and “cognitive dysfunction in SLE” was 0.87. These metrics highlight the strong interconnections between specific clinical and diagnostic research areas.

Analysis of temporal dynamics (**Figure 8C**) revealed the annual frequency trends for the nine core themes from 2006 to 2025. The “clinical manifestations of NPSLE” theme consistently remained the most frequent, demonstrating a sustained upward trajectory. Similarly, the annual frequencies of “NPSLE neuroimaging (MRI),” “cognitive dysfunction in SLE,” and “NPSLE cerebrospinal fluid/serum biomarkers” gradually increased over time. Independent temporal trajectories for each theme (**Figure 8D**) were evaluated using linear regression (**Supplementary Table 6**). Statistically significant upward trends were confirmed for “clinical manifestations of NPSLE” (Topic 0; R² = 0.498, P = 0.001), “autoimmune and clinical characteristics of SLE” (Topic 1; R² = 0.390, P = 0.004), “cognitive dysfunction in SLE” (Topic 6; R² = 0.468, P = 0.002), and “general characteristics of SLE” (Topic 7; R² = 0.439, P = 0.007). The trends for the remaining themes were not statistically significant (**Figure 8D**; **Supplementary Table 6**).

## 4 Discussion

### 4.1 Discussion based on bibliometric results

Within the context of systemic lupus erythematosus (SLE) research, the conceptualization of NPSLE has shifted from a rare, severe subtype to a recognized, prevalent complication. Our findings demonstrate sustained growth in publication volume over the past two decades, reflecting the field’s evolution from a niche area into an interdisciplinary research hotspot (13, 14). Early research primarily focused on disease definition and diagnostic criteria, whereas contemporary efforts have increasingly emphasized elucidating pathogenic mechanisms and optimizing targeted therapies. Geographically, China and the United States have emerged as the leading contributors (15). Parallel to this growing academic interest, the diagnostic approaches to NPSLE have evolved significantly. While early frameworks relied heavily on clinical symptoms, recent advancements have driven the development of a multidimensional diagnostic approach that integrates neuroimaging, fluid biomarkers, and clinical phenotyping. In particular, combining neuroimaging metrics with cognitive assessments has refined objective diagnostic algorithms for NPSLE (16, 17).

These diagnostic advancements have driven a parallel shift in therapeutic strategies, moving from broad-spectrum corticosteroids toward targeted immunotherapies (18). B-cell-targeted therapies remain a major research focus, and the neuroprotective efficacy of hydroxychloroquine has been increasingly recognized, establishing it as an evidence-based recommendation for NPSLE management (19, 20). Overall, the field has followed a logical continuum: establishing diagnostic frameworks (dominant in the early years), elucidating underlying pathogenic mechanisms, and most recently developing and optimizing targeted therapies. This data-driven trajectory mirrors the paradigm shift observed in research hotspots over time.

Furthermore, the heterogeneous development of NPSLE research is reflected in global collaboration networks. A distinct contrast exists between the two leading nations: China produces the highest volume of publications, while the United States leads in international collaboration and overall citation impact (21). Additionally, author productivity is highly concentrated among a small group of core researchers. While this concentration supports continuity in foundational research, it may also pose a barrier to entry for new investigators.

Based on the developmental trajectories and topic modeling results of the past two decades, several key frontiers in NPSLE research can be identified. Current hotspots focus on multimodal neuroimaging-informed assessment together with cognitive performance, multimodal biomarker discovery, and dedicated studies of pediatric NPSLE (22). Collectively, these trends indicate a broader shift in clinical research from qualitative phenotypic categorization to quantitative assessment, and from reactive management to proactive risk stratification and early intervention.

### 4.2 Integration of core evidence-based paradigms in the clinical management of NPSLE

Building on the bibliometric analysis, we conducted a systematic review and evidence synthesis of core clinical research, including 15 clinical trials, diagnostic accuracy studies, and observational cohorts (**Table 7**). This section evaluates the evidence base and existing gaps in NPSLE clinical management across three key dimensions: the evolution of diagnostic systems, the characterization of clinical risk factors, and the evidence supporting therapeutic strategies. This synthesis grounds the macroscopic bibliometric trends in clinical data and establishes a foundation for discussing pathogenesis and novel therapeutics.

**Table 7:** Summary of key clinical trials, diagnostic accuracy studies, and observational cohorts in NPSLE.

Study (year)	Study type	NPSLE definition/approach	Main result
Diagnostic studies			
Katsumata et al., 2010	PC	ACR (1999); Brain MRI	Any abnormal MRI: RR 1.7 (95% CI 1.1–2.7), p=0.016; Large lesions (≥10 mm): RR 3.7 (95% CI 2.9–4.7), p=0.0002
Vogelgesang et al., 1996	CS	Clinical attribution; CSF quinolinic acid	CSF level difference: p<0.014; CSF–serum correlation: p<0.003
Jung et al., 2012	CS	Neuropsychological battery; DTI	Total cognitive z-score difference: F = 13.2, p<0.001
Kovacs et al., 1995	Pilot	ACR (1999); Brain SPECT	SPECT positive rate: 85.7%; Combined with brain scan: 92.9%
Padovan et al., 2004	CS	ACR (1999); SPECT vs MRI	SPECT detection: 85%; Conventional MRI: 55%
Lee et al., 2012	CS	ACR (1999); PET/CT (MRI-negative)	Abnormal detection: 75%; Most affected: temporal/occipital (55% each)
Therapeutic studies			
Xie et al., 2021	Meta-analysis of RCTs	ICD coding; Belimumab safety	Psychiatric disorders: OR 0.89 (95% CI 0.64–1.23); All-cause mortality: OR 1.10 (95% CI 0.64–1.89)
23	RCT	IV cyclophosphamide vs methylprednisolone	Treatment response: RR 2.05 (95% CI 1.13–3.73)
Stojanovich et al., 2003	Pilot	ACR (1999); Low-dose cyclophosphamide + prednisone	Response rate: 96%; Between-group difference: p<0.05
Baca et al., 1999	Cohort	Clinical diagnosis; IVMP + IVCy (pediatric)	Overall improvement: 100%; Complete recovery: 60%; First-week improvement: 90%
Risk and outcome studies			
Palazzo et al., 2023	Phase III trial cohort	BILAG-2004; NPSLE flare risk	Male sex: HR 2.37 (95% CI 1.31–4.28); Baseline NP involvement: HR 5.91 (95% CI 3.86–9.06)
Monastero et al., 2000	CS	Clinical stratification; Cognitive impairment	Non-NP-SLE: 26.9%; NPSLE: 52.2%; Depression: independent predictor
Nowicka-Sauer et al., 2011	CS	Clinical stratification; Cognitive impairment	Overall: 57%; NP-SLE: 63.2%; Non-NP-SLE: 47.2%
Su et al., 2014	CS	ACR (1999); Autoantibodies vs oxidative stress	Anti-U1RNP: p=0.008; Anti-Sm: p=0.027; Anti-ribosomal P: p=0.028 (all negative correlations with glutathione)

NPSLE, neuropsychiatric systemic lupus erythematosus; SLE, systemic lupus erythematosus; CNS, central nervous system; ACR, American College of Rheumatology; BILAG, British Isles Lupus Assessment Group; RR, risk ratio; OR, odds ratio; CI, confidence interval; HR, hazard ratio; MRI, magnetic resonance imaging; SPECT, single-photon emission computed tomography; PET/CT, positron emission tomography/computed tomography; CSF, cerebrospinal fluid; IV, intravenous; RCT, randomized controlled trial; NR, not reported. Studies are organized by research design and presented in chronological order within each category. This table supports the evidence synthesis presented in Discussion section 4.2.

Current research indicates a shift in NPSLE diagnosis, moving from structural imaging alone to multimodal diagnostic workflows that incorporate functional imaging, reproducible neurocognitive testing, and biomarker readouts (24, 25). Regarding structural imaging, a prospective study by Katsumata et al. demonstrated that abnormal signals from large lesions (diameter ≥ 10mm) were exclusively observed in patients with central nervous system (CNS) involvement and significantly correlated with clinical outcomes (RR 3.7, 95% CI 2.9-4.7, P = 0.0002). However, the limited sensitivity of conventional MRI to minute lesions, diffuse alterations, and subclinical changes highlights the limitations of relying solely on structural imaging.

Functional imaging addresses these diagnostic limitations (26). Studies by Kovacs et al. and Padovan et al. reported that the abnormality detection rate of SPECT in NPSLE reached 85%, significantly outperforming conventional structural MRI. Furthermore, Lee et al. found that the positive detection rate of 18F-FDG PET/CT in MRI-negative patients was 75%, most frequently implicating the temporal, occipital, and frontal lobes. These findings demonstrate that functional imaging effectively mitigates the blind spots of conventional MRI, making it a valuable adjunctive diagnostic tool. Ultimately, combining functional and structural imaging improves diagnostic sensitivity and provides novel insights into the complex pathophysiology of NPSLE.

In terms of neurocognitive assessment, studies by Monastero et al. and Nowicka-Sauer et al. demonstrated a high overall prevalence of cognitive impairment (57%) in patients with SLE, regardless of overt neuropsychiatric manifestations. Notably, severe cognitive deterioration was exclusively observed in patients with overt NPSLE. This indicates that cognitive impairment is both a common complication of SLE and a key indicator of disease severity, providing a strong rationale for routine cognitive screening (27). Regarding biomarkers, Vogelgesang et al. provided initial evidence that cerebrospinal fluid quinolinic acid levels correlate significantly with SLE-associated neuropsychiatric manifestations. Concurrently, Su et al. characterized the relationship between anti-U1RNP, anti-Sm, and anti-ribosomal P antibodies and oxidative stress, identifying potential targets for the objective diagnosis and etiologic phenotyping of NPSLE (28, 29).

Although these investigations establish a foundation for a multidimensional diagnostic approach to NPSLE, the existing evidence base retains notable limitations. Consensus cutoff values and a harmonized interpretive framework across these diverse diagnostic modalities have not yet been established. Furthermore, symptom attribution relies heavily on clinical judgment; current attribution models yield positive predictive values (PPVs) ranging from 70% to 85% and lack an objective gold standard (30). Finally, specific populations—particularly pediatric NPSLE cohorts—face unique clinical challenges, including the absence of dedicated diagnostic criteria and frequent diagnostic delays.

### 4.3 Pathogenesis of NPSLE

**Figure 9:**
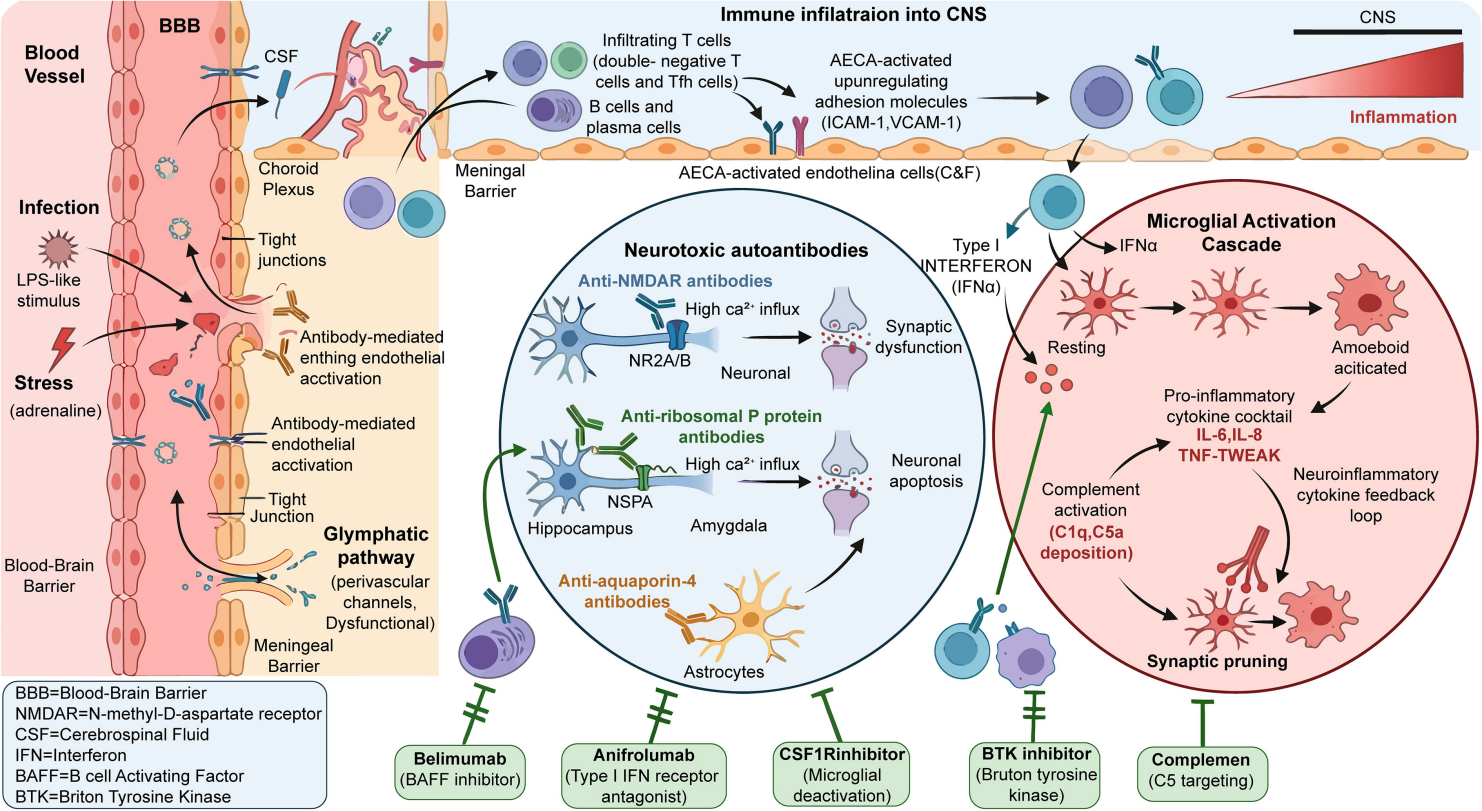
Schematic overview of the pathogenesis of neuropsychiatric systemic lupus erythematosus (NPSLE). The diagram illustrates the major pathogenic mechanisms involved in NPSLE. Disruption of the blood−brain barrier (BBB) and the blood−cerebrospinal fluid (CSF) barrier at the choroid plexus permits entry of pathogenic autoantibodies and immune cells into the central nervous system. Brain−reactive autoantibodies, such as anti−NMDAR and anti−ribosomal P antibodies, directly induce neuronal injury via excitotoxicity and synaptic dysfunction. Concurrently, activated microglia release pro−inflammatory cytokines (e.g., IL−6, TNF, IFNα) and contribute to complement−mediated synaptic pruning. Infiltrating T cells, B cells, and plasma cells further amplify neuroinflammation. Complement activation products (C1q, C5a) and type I interferon (IFNα) are key mediators that sustain the inflammatory cascade. The interplay among these mechanisms ultimately leads to neuronal damage, cognitive impairment, and other neuropsychiatric manifestations.

The pathogenesis of neuropsychiatric systemic lupus erythematosus (NPSLE) is highly heterogeneous, involving multiple factors and pathways; consequently, a unifying pathophysiological model has yet to be established (20). Nevertheless, current evidence indicates that the development of NPSLE involves a sequence of interconnected events that form a complex pathological network (**Figure 9**).

Central to this process is the disruption of the neuroimmune barrier. As the primary defense mechanism of the central nervous system (CNS), the compromise of the blood-brain barrier (BBB) is a critical prerequisite for the cerebral infiltration of pathogenic autoantibodies (31, 32). Antiphospholipid and anti-endothelial cell antibodies can activate vascular endothelial cells, upregulating the expression of adhesion molecules (e.g., ICAM-1 and VCAM-1) and increasing vascular permeability. Concurrently, “second-hit” events, such as infections or physiological stress, can induce transient BBB disruption, enabling circulating pathogenic factors to invade the CNS. Furthermore, the blood-cerebrospinal fluid (CSF) barrier, the meningeal barrier, and the recently characterized central lymphatic system also facilitate the permeation of immune cells and inflammatory mediators. Dysregulation of these barriers provides the structural basis for subsequent autoantibody influx and inflammatory cascades.

Following neuroimmune barrier disruption, brain-reactive autoantibodies enter the CNS and exert direct neurotoxic effects (33). Anti-NMDAR antibodies bind to the NR2A/B subunits of cerebral glutamate receptors, inducing excessive calcium influx and neuronal apoptosis via mechanisms analogous to glutamate excitotoxicity. Anti-ribosomal P protein antibodies target neuronal surface P antigens (NSPA) and perturb the synaptic modulatory functions of NMDARs; notably, within limbic regions such as the hippocampus and amygdala, this interference contributes to memory impairment and affective dysregulation. Conversely, anti-aquaporin-4 (AQP4) antibodies mediate astrocyte toxicity, which is implicated in NPSLE cohorts with concomitant demyelinating lesions. The pathogenicity of these autoantibodies has been validated in animal models, and adoptive transfer experiments confirm their direct pathogenic roles.

Concurrently, localized glial cells undergo aberrant activation. Type I interferon (IFNα)-mediated microglial activation is a primary pathogenic mechanism in NPSLE (34). Activated microglia secrete proinflammatory cytokines—including IL-6, IL-8, TNF, and TWEAK—and mediate abnormal synaptic loss via complement C1q-dependent synaptic pruning. This “innate immune memory” phenotype, imprinted on microglia following inflammatory stimulation, can persist long after the systemic inflammatory insult subsides. This enduring activation helps explain why NPSLE symptoms often occur independently of systemic SLE disease activity. Consequently, elevated CSF levels of IFNα, IL-6, CXCL10, and CCL2 have emerged as robust biomarkers of CNS-intrinsic inflammation.

Beyond cytokine-mediated inflammation, the complement cascade plays a pivotal role in NPSLE pathology (35, 36). Histopathological studies of NPSLE brain tissue demonstrate localized complement activation within the CNS. Complement components, particularly C5a and C1q, further disrupt BBB integrity, facilitate peripheral leukocyte infiltration, and amplify microglial activation. Although studies on the correlation between CSF and serum complement levels remain inconclusive, the emerging clinical efficacy of complement-targeted therapeutics (e.g., eculizumab) in refractory NPSLE supports the pathogenic role of complement activation in specific endotypes.

Alongside these innate immune mechanisms, adaptive immunity is significantly involved in NPSLE pathophysiology (37). T cells (including double-negative T cells and T follicular helper cells), B cells, and plasma cells can infiltrate the brain parenchyma via perivascular spaces, predominantly breaching the CNS through the blood-CSF barrier at the choroid plexus. These infiltrating cells can synthesize pathogenic autoantibodies intrathecally, further activating glia and sustaining a localized neuroinflammatory environment that exacerbates neurotoxicity.

### 4.4 Recent advances in NPSLE therapeutics: from broad-spectrum immunosuppression to targeted interventions

Therapeutic research in NPSLE has transitioned from reliance on broad-spectrum corticosteroids toward optimized immunosuppressive regimens and targeted therapies (23). Regarding conventional immunosuppression, a randomized controlled trial by Barile-Fabris et al. demonstrated that the clinical response to intravenous cyclophosphamide in severe NPSLE significantly exceeded that of intravenous methylprednisolone (RR 2.05, 95% CI 1.13–3.73). This finding provides strong evidence for its use as a first-line treatment in severe cases. Furthermore, studies by Ramos et al. and Stojanovich et al. showed that combining low-dose intravenous cyclophosphamide with prednisone yielded a 96% response rate alongside a favorable tolerability profile. This regimen offers an effective, lower-toxicity alternative for patients intolerant to high-dose immunosuppressive therapy. Collectively, these studies establish cyclophosphamide as a central component in the clinical management of NPSLE (38).

Regarding biologic agents, a meta-analysis by Xie et al. demonstrated that belimumab does not significantly increase the risk of psychiatric disorders (OR 0.89, 95% CI 0.64–1.23) or all-cause mortality (OR 1.10, 95% CI 0.64–1.89) in SLE patients with concurrent neuropsychiatric symptoms, thereby supporting its safety in clinical practice (39, 40). This evidence gap is largely attributable to the frequent exclusion of patients with active NPSLE from major SLE registration studies. Consequently, mainstream targeted therapeutics—such as rituximab and anifrolumab—lack dedicated, high-quality, randomized controlled data specific to the NPSLE population (41, 42). The disparity between the clinical burden of NPSLE and the low quality of evidence for its treatment presents a critical gap that must be addressed in the rigorous design of future clinical trials. The evolution of therapeutic strategies and the core evidence are summarized in **Table 8**. Despite these findings, the overall quality of evidence for novel targeted NPSLE therapies remains limited (43).

**Table 8:** Emerging therapeutic strategies for NPSLE: from broad-spectrum immunosuppression to targeted interventions.

Strategy (target/pathway)	Example interventions	Evidence snapshot	Key gaps/next steps
B-cell depletion (CD20)	Rituximab	Effective for severe inflammatory NPSLE in observational cohorts/open-label trials	No high-quality NPSLE-specific RCT evidence
B-cell depletion (CD19)	CD19 CAR-T	Long-term drug-free remission in refractory SLE; compassionate use improved neuro symptoms/lesions (no ICANS)	Need large multicenter trials; long-term safety unknown
B-cell signaling (BTK)	Evobrutinib; Orelabrutinib	Orelabrutinib showed dose-dependent efficacy trend in phase Ib/IIa; improved cognition in mouse models	Evobrutinib failed phase II endpoint; phase IIb trial ongoing
Autoantibody neutralization	FISLE-412 peptide	Favorable tolerability; delayed SLE onset in NZB/W F1 mice	Only preclinical validation; no human trials initiated
Type I IFN signaling (IFNAR)	Anifrolumab	Approved for moderate-to-severe SLE	Active severe NPSLE excluded from pivotal trials; efficacy unvalidated
Type I IFN signaling (BDCA2)	Litifilimab	In phase II clinical development for SLE	No NPSLE-specific clinical data available
Intracellular signaling (TYK2)	Deucravacitinib	Significant efficacy in phase II SLE trial; advanced to phase III	Limited blood-brain barrier (BBB) penetration
Intracellular signaling (JAK)	Baricitinib; Upadacitinib	Under clinical evaluation for SLE	Limited CNS penetrance; NPSLE value poorly defined
CNS neuroprotection (ACEi)	Captopril; Lisinopril	Improved memory in anti-NMDAR mouse model; phase II trial ongoing (NCT04486118)	Trial not completed; clinical efficacy unconfirmed
BBB preservation (AHR axis)	Dietary intervention; flavonoids	Preclinical data support improved BBB integrity via gut microbiota modulation	Mechanistically complex; no clinical validation data

NPSLE, neuropsychiatric systemic lupus erythematosus; SLE, systemic lupus erythematosus; CNS, central nervous system; BBB, blood-brain barrier; CAR-T, chimeric antigen receptor T cell; BTK, Bruton’s tyrosine kinase; ACEi, angiotensin-converting enzyme inhibitor; JAK, Janus kinase; TYK2, tyrosine kinase 2; ICANS, immune effector cell-associated neurotoxicity syndrome; BILAG-BR, British Isles Lupus Assessment Group Biologics Register; RCT, randomized controlled trial. Therapeutic strategies are categorized into three major mechanisms: modulation of B cell activity and autoantibody production, attenuation of systemic inflammatory burden, and direct intervention in CNS pathological processes. This table summarizes the key advances and current limitations discussed in Section 4.4.

### 4.5 Management of NPSLE and future perspectives

Through a comprehensive bibliometric analysis of 1,569 NPSLE-related publications from 2006 to 2025, this study delineates the 20-year academic trajectory and evolving research paradigms within the field (44). Integrating recent advances in pathogenesis, diagnostic technologies, and therapeutics, it is evident that NPSLE clinical management is transitioning from empirical, symptom-directed management toward mechanism-informed, stratified interventions. However, this transition is constrained by incomplete diagnostic agreement, substantial gaps in evidence-based medicine, and the complex heterogeneity of NPSLE pathogenesis. Future research must address these challenges by optimizing diagnostic frameworks, stratifying treatment strategies, standardizing clinical research, advancing translational medicine, and fostering global collaboration. These efforts are essential to improve clinical management, long-term prognoses, and patient quality of life.

Regarding the diagnostic framework, constructing a multidimensional approach with agreed-upon interpretive rules is critical. Specifically, this requires integrating clinical phenotypes, multimodal imaging, fluid biomarkers, and standardized neurocognitive scales. This integration necessitates optimizing symptom attribution models and interpretative thresholds, alongside developing dedicated diagnostic criteria for specific populations, such as pediatric cohorts. Particular emphasis should be placed on establishing interpretable links between neuroimaging metrics and cognitive performance, together with clinically actionable decision rules to provide quantitative benchmarks for clinical practice.

In the clinical trial domain, initiating multicenter, large-scale randomized controlled trials (RCTs) dedicated exclusively to NPSLE is urgently needed. The systematic exclusion of patients with active NPSLE from pivotal SLE trials must be addressed. It is essential to evaluate the efficacy and safety of novel targeted therapeutics—such as CAR-T cell therapy, BTK inhibitors, Type I interferon pathway inhibitors, and CNS-penetrant ACE inhibitors—specifically within NPSLE cohorts (45). A critical priority is establishing a standardized clinical endpoint evaluation system. For cognitive dysfunction—a highly prevalent yet challenging endpoint to quantify—exploring imaging or fluid biomarkers as surrogate endpoints is essential to reduce trial complexity and improve outcome comparability.

In translational research, efforts to elucidate NPSLE pathogenesis must be expanded. Key objectives include deciphering the mechanisms of neuroimmune barrier disruption, identifying how microglial “innate immune memory” drives chronic injury, and clarifying the regulatory dynamics of the gut-brain axis. Concurrently, optimizing animal models and applying multi-omics strategies are crucial to translating novel interventions—such as blood-brain barrier neuroprotection and microglial modulation—into clinical practice. Furthermore, tailored investigations into distinct subsets, notably pediatric NPSLE, require increased attention.

Synergistic advancement across diagnostics, clinical trials, and translational research is critical to resolving the disparity between the high disease burden of NPSLE and the low quality of therapeutic evidence. Ultimately, these efforts should facilitate the transition from empiric management to mechanism-driven, precision interventions.

### 4.6 Limitations

Several methodological limitations should be considered when interpreting these findings. First, to ensure standardized citation metrics and cross-study comparability, this analysis was restricted to English-language publications. While this criterion maintains methodological consistency, it inevitably excludes relevant research published in other languages, such as Chinese, Japanese, or Korean. Although these studies hold contextual value, their integration into the global citation network is generally more limited.

Furthermore, inherent discrepancies exist between the Web of Science Core Collection (WoSCC) and Scopus regarding indexing scope, update frequency, and document types. Specifically, WoSCC offers a more extensive historical archive and a stable citation structure, whereas Scopus provides a broader indexing scope and faster update cycles. To reconcile these differences, cross-database deduplication and metadata standardization protocols were implemented. Nevertheless, subtle variations in indexing logic may still marginally affect the precise mapping of institutional affiliations and collaborative networks. Additionally, the complete standardization of author and institutional names is limited by the capabilities of current bibliometric software. Moreover, because the present study was based on bibliometric metadata (e.g., authors, affiliations, countries, and citations) rather than structured extraction from full texts, we were unable to systematically quantify institution-specific responsibilities in multicenter studies (e.g., patient recruitment, biomarker testing, and imaging assessment). Consequently, the publication output at the country and institutional levels may not be directly comparable because different counting conventions were applied.

However, given the large sample size and the extensive timeframe evaluated, these confounding variables are unlikely to meaningfully alter the overarching trends observed. Ultimately, bibliometric analyses provide a retrospective overview of academic progress and cannot replace prospective clinical trials or foundational mechanistic studies. Within this context, the present study provides an objective, quantitative framework for understanding the evolutionary trajectory of NPSLE research.

## 5 Conclusion

Based on a comprehensive bibliometric analysis of 1,569 publications retrieved from the Web of Science Core Collection and Scopus databases, integrated with evidence from core clinical trials, this study systematically delineates the evolutionary trajectory of neuropsychiatric systemic lupus erythematosus (NPSLE) research from 2006 to 2025, including diagnostic and therapeutic advancements and central bottlenecks. Our findings reveal a robust average annual growth rate of 6.04% in publication volume over the past two decades, with China and the United States emerging as the preeminent global contributors. Importantly, the epicenter of research interest has undergone a profound paradigm shift from rudimentary disease definition and foundational diagnosis toward increasingly precision-oriented therapeutics and deeper mechanistic exploration. Concurrently, diagnostic frameworks are rapidly evolving into multidimensional, highly integrated paradigms, while therapeutic strategies are experiencing a definitive shift from broad-spectrum immunosuppression to precision-targeted interventions.

Despite these advances, the field remains critically encumbered by major systemic challenges, including the lack of consensus diagnostic cutoff values and a dearth of robust evidence-based support for targeted therapeutics. Consequently, NPSLE continues to face a persistent developmental paradox characterized by high clinical prevalence alongside comparatively low-quality evidence. Moving forward, it is essential to prioritize the construction of standardized diagnostic and evaluative ecosystems, initiate large-scale multicenter clinical trials exclusively dedicated to patients with NPSLE, and further deepen translational research into underlying pathogenesis. Addressing these priorities will catalyze the transition of NPSLE clinical management from empiric symptomatic treatment to mechanism-driven, precision interventions, thereby improving long-term patient outcomes and overall functional status.

## Data Availability

The original contributions presented in the study are included in the article/**Supplementary Material**. Further inquiries can be directed to the corresponding author.
