# Supplementary material for: From empirical treatment to precision intervention: a multi-database bibliometric analysis of neuropsychiatric systemic lupus erythematosus (2006–2025)
Source: Front Immunol. 2026 Jun 3;17:1846304. doi: 10.3389/fimmu.2026.1846304 (PMC13272480; doi:10.3389/fimmu.2026.1846304)
Supplement: Supplementary file 1 [file Table1.docx]

**Supplementary Table 1. Search strategies employed in WoSCC and Scopus databases**

| **Database** | **Search Strategy** | **Search Date** | **Filtering Conditions** | **Field Code** |
| --- | --- | --- | --- | --- |
| WOSCC | TS=("NPSLE" OR "neuropsychiatric lupus" OR ("neuropsychiatric manifestation*" AND ("systemic lupus erythematosus" OR "SLE")) OR ("central nervous system lupus" OR "CNS lupus")) | The literature search was conducted on January 15, 2026. | English-language publications from January 1, 2006, to December 31, 2025, including Articles and Review Articles. | Topic Search |
| Scopus | TITLE-ABS-KEY ("NPSLE" OR "neuropsychiatric lupus" OR ( "neuropsychiatric manifestation*" AND ( "systemic lupus erythematosus" OR "SLE" ) ) OR ( "central nervous system lupus" OR "CNS lupus" )) AND PUBYEAR > 2005 AND PUBYEAR < 2026AND ( DOCTYPE("ar") OR DOCTYPE("re") ) AND LANGUAGE("English") | The literature search was conducted on January 15, 2026. | English-language publications from January 1, 2006, to December 31, 2025, including Articles and Review Articles. | ABS, Title/Abstract/Keywords |
| PubMed | “Lupus Erythematosus, Systemic”[Mesh] AND ( “Nervous System Diseases”[Mesh] OR “Mental Disorders”[Mesh] ) | The literature search was conducted on January 15, 2026. | Clinical Trials from January 1, 1990, to December 31, 2025. | MeSH terms |

Abbreviations： WoSCC, Web of Science Core Collection; TS, topic search (searches title, abstract, and keywords); TITLE-ABS-KEY, search field for title, abstract, and keywords; MeSH, Medical Subject Headings (standardized indexing terms used by PubMed); SLE, systemic lupus erythematosus; CNS, central nervous system; DOCTYPE, document type; "ar", article; "re", review. Notes: This table presents the detailed search strategies used to retrieve neuropsychiatric systemic lupus erythematosus (NPSLE) research literature from three databases: Web of Science Core Collection (WoSCC) and Scopus: Searches were conducted for the period 2006–2025 to generate the bibliometric analysis dataset. PubMed: A separate search was conducted for the period 1990–2025 to identify core clinical studies for systematic evidence synthesis. All searches were performed on January 15, 2026.

**Supplementary Table 2. Top 20 high-frequency keywords and their occurrence counts**

| **Keywords** | **Count** |
| --- | --- |
| systemic lupus erythematosus | 886 |
| neuropsychiatric lupus | 396 |
| human | 258 |
| disease | 217 |
| prevalence | 213 |
| autoantibody | 205 |
| manifestations | 193 |
| neuropsychiatric systemic lupus erythematosus | 187 |
| female | 174 |
| classification | 165 |
| erythematosus | 163 |
| neuropsychiatric manifestations | 161 |
| adult | 158 |
| association | 152 |
| central nervous system | 150 |
| antibody | 143 |
| diagnosis | 143 |
| lupus erythematosus | 135 |
| cyclophosphamide | 120 |
| brain | 113 |

Abbreviations: n, frequency of keyword occurrence. Keyword analysis was performed using CiteSpace (v6.2.R1) based on the combined dataset from WoSCC and Scopus. Notes: This table lists the top 20 most frequently occurring keywords in the combined dataset of NPSLE research (2006–2025). Keywords were extracted from the titles and abstracts of included publications.

**Supplementary Table 3. Top 20 authors by citation count with publication output and total link strength (TLS)**

| **Author** | **Citations** | **Total Link Strength** |
| --- | --- | --- |
| Bertsias, George K. | 1831 | 60 |
| Putterman, Chaim | 1666 | 53 |
| Shoenfeld, Yehuda | 1513 | 28 |
| Diamond, Betty | 1123 | 29 |
| Steup-Beekman, Gerda M. | 904 | 96 |
| Appenzeller, Simone | 907 | 76 |
| Cervera, Ricard | 847 | 5 |
| Volpe, Bruce T. | 794 | 19 |
| Hirohata, Shunsei | 788 | 27 |
| Stock, Ariel D. | 774 | 19 |
| Zandman-Goddard, Gisele | 755 | 9 |
| Hanly, John G. | 920 | 11 |
| Chapman, Joab | 688 | 25 |
| Huizinga, Tom W. J. | 684 | 86 |
| Kowal, Czeslawa | 679 | 15 |
| Huerta, Patricio T. | 675 | 15 |
| Van Buchem, Mark A. | 666 | 50 |
| Kozora, Elizabeth | 662 | 8 |
| Wen, Jing | 597 | 30 |
| Arinuma, Yoshiyuki | 569 | 33 |

Abbreviations: TC, total citations; Pub, total publications; TLS, total link strength (co-authorship network). Authors are ranked by total citation count. Network metrics were calculated using VOSviewer (v1.6.20). Notes: This table presents the top 20 authors in NPSLE research (2006–2025) ranked in descending order of total citations. Network metrics were calculated using VOSviewer (v1.6.20).

**Supplementary Table 4. Top 20 countries by citation count with publication output and total link strength (TLS)**

| **Country** | **Citations** | **Total Link Strength** |
| --- | --- | --- |
| United States | 12662 | 258 |
| Italy | 6548 | 109 |
| United Kingdom | 6115 | 161 |
| Greece | 4791 | 78 |
| Netherlands | 4468 | 97 |
| Spain | 4205 | 109 |
| Canada | 3834 | 140 |
| Sweden | 3813 | 120 |
| Germany | 3769 | 90 |
| China | 3767 | 49 |
| Japan | 3452 | 41 |
| Israel | 3020 | 68 |
| Austria | 2852 | 48 |
| France | 2646 | 65 |
| Czech Republic | 2362 | 32 |
| Belgium | 2346 | 54 |
| Brazil | 2140 | 44 |
| Denmark | 1919 | 74 |
| Cyprus | 1645 | 19 |
| Mexico | 1345 | 72 |

Abbreviations: TC, total citations; Pub, total publications; TLS, total link strength (international collaboration network). Notes: This table presents the top 20 countries in NPSLE research (2006–2025) ranked in descending order of total citations. Network metrics were calculated using VOSviewer (v1.6.20).

**Supplementary Table 5. Top 20 institutions by citation count with publication output and total link strength (TLS)**

| **Organization** | **Citations** | **Total Link Strength** |
| --- | --- | --- |
| Tel Aviv Univ | 2184 | 18 |
| Leiden Univ | 1892 | 3 |
| Albert Einstein Coll Med | 1657 | 31 |
| Dalhousie Univ | 1464 | 28 |
| Sheba Med Ctr | 1370 | 14 |
| Univ Crete | 1326 | 33 |
| Univ Toronto | 1148 | 17 |
| Kitasato Univ | 964 | 17 |
| Univ Campinas | 899 | 14 |
| Univ Ferrara | 822 | 14 |
| INCMNSZ | 815 | 3 |
| Pontificia Univ Católica Chile | 811 | 2 |
| Feinstein Inst Med Res | 790 | 10 |
| Univ Colorado | 698 | 14 |
| Teikyo Univ | 698 | 11 |
| Johns Hopkins Univ | 650 | 13 |
| UCL | 618 | 7 |
| FORTH | 589 | 23 |
| QEII Hlth Sci Ctr | 570 | 29 |
| Northwestern Univ | 532 | 19 |

Abbreviations: TC, total citations; Pub, total publications; TLS, total link strength (institutional collaboration network). Institutions are ranked by total citation count.

**Supplementary Table 6. Linear regression statistics (R² and P-values) for temporal trends of BERTopic themes**

| **Topic** | **Linear_r2** | **Linear_p** |
| --- | --- | --- |
| 0 | 0.497635257 | 0.000511845 |
| 1 | 0.389625736 | 0.004283376 |
| 2 | 0.170082119 | 0.070761239 |
| 3 | 0.145188343 | 0.097410355 |
| 4 | 0.245313401 | 0.031064627 |
| 5 | 0.05055329 | 0.340563322 |
| 6 | 0.467580132 | 0.002471563 |
| 7 | 0.439035703 | 0.007106714 |
| 8 | 0.057219938 | 0.372250367 |

Abbreviations: R², coefficient of determination; P, P-value for the slope of the linear regression.

Statistically significant trends (P < 0.05) are highlighted in bold. Temporal trends were analyzed using linear regression on annual topic frequencies from 2006 to 2025.

**Supplementary Table 7. Inclusion and Exclusion Criteria for Core Clinical Studies of Neuropsychiatric Systemic Lupus Erythematosus**

| Category | Inclusion Criteria | Exclusion Criteria |
| --- | --- | --- |
| General Criteria | 1. Publication period: January 1, 1990 to May 1, 20252. Language: Full-text articles published in English3. Study type: Clinical Trial as defined by PubMed4. Study population: Human subjects with confirmed diagnosis of systemic lupus erythematosus (SLE)5. Data completeness: Containing extractable study design, core results, and statistical values | 1. Articles published in languages other than English2. Duplicate publications (only the most recent version with the most complete data was retained for the same study)3. Conference abstracts, letters, commentaries, expert consensuses, or guideline documents4. Basic experimental studies (cell or animal experiments)5. Articles with missing core research data that could not be extracted |
| Specific Criteria for Core Clinical Studies | 1. Research topics directly related to neuropsychiatric systemic lupus erythematosus (NPSLE), covering any of the following directions: - Diagnostic tools and assessment methods for NPSLE - Clinical characteristics, risk factors, and prognosis of NPSLE - Efficacy and safety of treatment regimens for NPSLE - Clinical exploration of biomarkers and pathological mechanisms related to NPSLE2. Explicit adoption of internationally recognized SLE diagnostic criteria (mainly the 1997 American College of Rheumatology (ACR) classification criteria)3. Including subgroup analysis of patients with NPSLE or studies specifically targeting the NPSLE population | 1. Studies focusing only on other system involvements of SLE (renal, cutaneous, articular, etc.)2. Neuropsychiatric manifestations unrelated to SLE (caused by other etiologies)3. Studies that did not separately report subgroup data of NPSLE patients4. Case reports or small case series with a sample size < 55. Articles with serious flaws in study design or obvious contradictions in data |

Abbreviations. NPSLE, neuropsychiatric systemic lupus erythematosus; SLE, systemic lupus erythematosus; ACR, American College of Rheumatology; NLM, National Library of Medicine. Notes. This table presents the inclusion and exclusion criteria used for the systematic evidence synthesis of core clinical studies in NPSLE. The time window for clinical studies was extended back to 1990 to include foundational studies that established the current diagnostic and therapeutic framework for NPSLE, while the bibliometric analysis in the main text focused on the period 2006–2025 to reflect modern research trends. PubMed’s "Clinical Trial"[pt] tag is a hierarchical parent label that includes interventional clinical trials, diagnostic accuracy studies, and high-quality observational clinical studies as defined by the National Library of Medicine (NLM) classification system. All included studies were indexed under this official tag. The sample size threshold of <5 was set to exclude anecdotal evidence (case reports and very small case series) and maintain minimal statistical reliability of the included results. NPSLE diagnosis was based on the 1999 ACR case definitions or explicitly described standardized attribution criteria that excluded other etiologies of neuropsychiatric manifestations.
